# Lipid-Lowering Effects of Lotus Leaf Alcoholic Extract on Serum, Hepatopancreas, and Muscle of Juvenile Grass Carp via Gene Expression

**DOI:** 10.3389/fphys.2020.584782

**Published:** 2020-12-04

**Authors:** Junpeng Yao, Pengcheng Hu, Yanhong Zhu, Yingyan Xu, Qingsong Tan, Xufang Liang

**Affiliations:** College of Fisheries, Huazhong Agricultural University/Key Laboratory of Freshwater Animal Breeding, Ministry of Agriculture, China/Hubei Provincial Engineering Laboratory for Pond Aquaculture/Engineering Research Center of Green development for Conventional Aquatic Biological Industry in the Yangtze River Economic Belt, Ministry of Education, Wuhan, China

**Keywords:** *Ctenopharyngodon idellus*, plant extract, molecular pathway, lipogenesis, lipid catabolism, lipid transportation

## Abstract

Compared with wild grass carp (*Ctenopharyngodon idellus*), intensively cultured fish displayed disordered lipid metabolism, showing excess lipid deposition in the hepatopancreas and muscle. Lotus leaf prevents fat accumulation in humans and may have similar effects on fish. This study explored the regulatory mechanisms by which the dietary addition of an alcoholic extract of lotus leaf (AELL) reduced lipid deposition in the hepatopancreas and muscle of juvenile grass carp. The fish (average initial weight: 34.00 ± 0.40 g) were fed four experimental diets containing different AELL levels (0, 0.07, 0.14, and 0.21%) for 8 weeks. Serum components, lipid droplet size, triacylglycerol (TAG) content, enzymatic activities, and mRNA levels of genes related to lipid metabolism in the hepatopancreas and muscle were analyzed. The results show that dietary AELL supplementation significantly reduced the TAG content and lipid droplet area in the histological sections as well as the fatty acid synthase (FAS) activity in both the hepatopancreas and muscle but enhanced the activities of lipoprotein lipase (LPL) and carnitine palmitoyltransferase I (CPT1) in both tissues. In addition, dietary AELL supplementation decreased the mRNA expression of genes involved in fatty acid uptake (*cd36*, *fatp1/fatp4/fatp6*, *fabp10/fabp11*, *acsl1/acsl4*) and *de novo* lipid synthesis (*pgd*, *g6pd*, and *fasn*) as well as the transcription factors *pparg* and *srebf1* in the hepatopancreas and muscle but increased the mRNA levels of genes relating to lipid catabolism (*cpt1a*, *lipe*, *pnpla2*, *lpl*), lipid transportation (*apob*), and the transcription factor *ppara* in both tissues. In conclusion, dietary AELL supplementation reduced lipid accumulation in the hepatopancreas and muscle by affecting the gene expression of proteins with known effects on lipid metabolism in juvenile grass carp.

## Introduction

Grass carp (*Ctenopharyngodon idellus*) is an important commercial freshwater fish in high demand within China (FAO, 2018). To meet market needs, modern intensive aquaculture is based on formulated feeds that promote fish growth and increase grass carp production (Zhao et al., 2018). However, the formulated feeds induce lipid metabolism disorders, causing excess fat deposition in the fish (especially in the hepatopancreas and muscle), resulting in physiological impairment and the degradation of flesh quality (Wang et al., 2018; Zhao et al., 2018). Thus, maintaining lipid homeostasis has become an important topic in modern aquaculture practice (Gui and Zhu, 2012).

In recent years, plant extracts have been used as functional and environmentally friendly feed additives in aquaculture due to their promotion of appetite and growth in fish and their ability to control diseases (Hoseinifar et al., 2020). Some plant extracts also show the potential to prevent fat deposition by regulating energy intake and lipid metabolism (Song et al., 2016; Kim et al., 2017). Lotus (*Nelumbo nucifera*) is a perennial aquatic herb that is widely distributed throughout eastern Asia (Kim et al., 2009). The seeds and flowers of the lotus, as well as extracts of the rhizomes and leaves, are widely used in traditional medicine due to their pharmacological actions (Mukherjee et al., 2009). Investigation of phytochemicals in the lotus shows that dry lotus leaves are rich in 11 flavonoids (up to 84.21 mg g^–1^) and 8 alkaloids (up to 2.132 mg g^–1^) as well as other active ingredients (Ahn et al., 2013; Zhao and Deng, 2013). The flavonoids in the alcoholic extract of the lotus leaf are a group of polyphenolic compounds that mainly includes kaempferitrin, hyperoside, astragalin, phloridzin, and quercetin (478.51, 232.74, 12.62, 5.67, and 0.49 mg g^–1^, respectively) (Chen et al., 2020). These have important physiological functions, including anti-inflammatory, anti-allergic, anti-obesity, and antioxidant activities (Agrawal, 2011; Ahn et al., 2013). The alkaloids in lotus leaf extracts also have other beneficial health effects, including alleviating hyperlipemia, resisting karyokinesis (Ma et al., 2010), and antimicrobial and antifungal properties (Agnihotri et al., 2008). The biological potential to lower blood lipid and the anti-obesity effects of lotus leaf extract have been shown in humans (Lee et al., 2015; Zhang et al., 2015) and rats (Su et al., 2015; Ding et al., 2017) and indicate that dietary lotus leaf extract also may have the potential to suppress excess lipid accumulation in fish.

Lipid metabolism in tissues consists of a series of biological processes, including circulating lipid uptake, lipid transportation, *de novo* fatty acid synthesis (lipogenesis), and lipid catabolism via β-oxidation (Chen et al., 2014), which results in lipid deposition or depletion. The pathways of lipid metabolism have been well defined in mammals and are considered to be similar in fish (Zheng et al., 2015). Lipoprotein lipase (LPL) hydrolyzes lipoprotein triacylglycerol (TAG) to supply free fatty acids for lipid metabolism (Nilsson-Ehle et al., 1980). The fatty acid transporters, such as fatty acid translocase/CD36, cytoplasmic fatty acid binding proteins (FABPs), and fatty acid transport proteins (FATPs), are indispensable for fatty acid absorption (Sclafani et al., 2007). Fatty acid synthase (FAS) (encoded by *fasn*), 6-phosphogluconate dehydrogenase (PGD), glucose 6-phosphate dehydrogenase (G6PD), and acetyl-CoA carboxylase alpha (ACC) (encoded by *acaca*) play important roles in lipogenesis (Tan et al., 2019). Carnitine palmitoyltransferase I (CPT1) (encoded by *cpt1a*) catalyzes the conversion of fatty acid CoAs into fatty acid carnitines, a key process of long-chain fatty acid oxidation in the mitochondrial matrix (Kerner and Hoppel, 2000). Several transcription factors, such as peroxisome proliferator activated receptor alpha and gamma (PPARA and PPARG) and sterol regulatory element binding transcription factor 1 (SREBF1), are considered to play regulatory roles in lipid metabolism via orchestrating the gene transcription of the enzymes in these pathways (Duque-Guimarães et al., 2009; Minghetti et al., 2011). In addition, lipid metabolism involves several other functional proteins in different tissues. In the liver, apolipoprotein B (APOB) plays a pivotal role in very low-density lipoprotein (VLDL) assembly and secretion (Tanoli et al., 2004), and in muscle cells, the low-density lipoprotein receptor-related protein 1 (LRP1) or very low density lipoprotein receptor (VLDLR) is important in mediating the endocytosis of these lipoproteins (mainly VLDL) (Perman et al., 2011).

The regulatory mechanism of dietary lotus leaf extract on lipid metabolism is well defined in mammals, such as humans (Lee et al., 2015; Zhang et al., 2015) and rats (Su et al., 2015; Ding et al., 2017). Very limited studies regarding the use of lotus leaf extract in aquafeeds are reported, and only its growth-promoting effects have been investigated (Munglue, 2015). Our previous study reports that lotus leaf alcohol extract promotes the growth of grass carp but also decreases the body fat content of the fish (Zhu et al., 2019). However, the mechanism by which lotus leaf extract regulates fat deposition is unclear. The aim of the present study was to investigate the potential of lotus leaf extract to reduce fat deposition in the hepatopancreas and muscle of grass carp and determine the affected pathway(s) in order to provide better insight into the nutritional regulation of fish lipid metabolism.

## Materials and Methods

All procedures were approved by the Institutional Animal Care and Use Committee of Huazhong Agricultural University (Wuhan, China) for laboratory animal use. Culturing of fish was performed according to the common Organization for Economic Cooperation and Development protocol for fish.

### Diet Preparation and Feeding Trial

The preparation [including alcoholic extract of lotus leaf (AELL)], ingredients, and proximate composition of the experimental diets (Table 1) were the same as described by Zhu et al. (2019). In brief, four experimental diets of four groups, namely AELL0 (control), AELL7, AELL14, and AELL21, were formulated by supplementing four levels of AELL (0, 0.07, 0.14, and 0.21%, respectively; that is, the flavonoids of four experimental diets were 0, 0.26, 0.57, and 0.97 g/kg feed, respectively) into a basal diet (30% crude protein and 7.8% crude lipid). All the ingredients were ground into powder, sieved through a 60-mesh screen, blended in a mixer to homogenize thoroughly, and then pelleted (diameter: 2.0 mm, length: 6 mm) in a laboratory pelletizer. The pellets of the diets were air-dried and stored at −20°C for later use. The animal feeding trials were the same as described in Zhu et al. (2019). Experimental fish were acclimated to the culture conditions for 2 weeks. Afterward, 360 uniform-sized individuals (initial body weight: 34.00 ± 0.40 g) were randomly assigned to 12 300-L circular fiberglass tanks with 30 fish per tank. During the 8-week feeding trial, the fish were fed with the four experimental diets twice daily (8:30 am and 16:00 pm, respectively) to apparent satiation on the basis of visual observation with each diet randomly distributed to the 3 tanks. During the trial, fish were reared under a natural daylight cycle of 12 h:12 h (light:dark) with continuous aeration and running water (0.5 L min^–1^). During the trial, the water temperature ranged from 24 to 29°C and the pH was 6.7–7.7. The dissolved oxygen level was more than 5 mg L^–1^, and ammonia nitrogen was less than 0.3 mg L^–1^.

**TABLE 1:** Diet formulation and composition of the experimental diets (%).

Ingredient	Dietary lotus leaf extract levels			
	AELL0	AELL7	AELL144	AELL21
Fish meal	1.50	1.50	1.50	1.50
Soybean meal	30.00	30.00	30.00	30.00
Cottonseed meal	6.00	6.00	6.00	6.00
Rapeseed meal	30.00	30.00	30.00	30.00
Wheat flour	20.50	20.50	20.50	20.50
Soybean oil	4.00	4.00	4.00	4.00
Choline chloride (50%)	0.15	0.15	0.15	0.15
Ethoxyquin (30%)	0.05	0.05	0.05	0.05
Monocalcium phosphate	1.60	1.60	1.60	1.60
Microcrystalline cellulose	2.20	2.13	2.06	1.99
Salt	0.40	0.40	0.40	0.40
Compound premix^a^	1.00	1.00	1.00	1.00
AELL	0.00	0.07	0.14	0.21
Bentonite	2.50	2.50	2.50	2.50
Mould inhibitor	0.10	0.10	0.10	0.10
***Nutrient content* (%)**				
Crude protein	30.12	29.87	29.76	29.74
Crude lipid	7.98	7.85	7.81	7.82
Ash	4.71	4.66	4.58	4.61
Moisture	8.91	8.87	8.81	9.13
Flavonoids (g/kg)	0.00	0.26	0.57	0.97

*^*a*^Per kilogram of compound premix containing vitamin A, 300,000 IU; vitamin D_3_, 100,000 IU; vitamin E, 1.2 g; vitamin k_3_, 0.5 g; vitamin B_1_, 0.5 g; vitamin B_2_, 0.7 g; vitamin B_6_, 0.6 g; vitamin B_12_, 0.0015 g;. vitamin B_5_, 2.5 g; vitamin C, 10 g; folic acid 0.15 g, D-calcium pantothenate 1.8 g, biotin 0.003 g, inositol 8 g, magnesium 10 g, manganese 2 g, iron 12 g, zinc 5 g, copper 0.4 g, iodine 0.1 g, cobalt 0.03 g, selenium 0.01 g.*

### Sample Collection

At the end of the feeding trial, all fish were fasted for 24 h before the fish in each tank were rapidly anesthetized with MS-222 (Sigma, United States) (75 mg/L). Blood samples were taken through the caudal vein from 3 randomly selected fish in each tank and stored on crushed ice. Serum was obtained from the blood samples by centrifugation (3000 × *g*, 10 min, 4°C) after clotting at 4°C, aliquoted, and stored at −80°C until subsequent analyses. The hepatopancreas and white muscle tissues were dissected from the 3 fish, aliquoted, and immersed into liquid nitrogen immediately before storage at −80°C for gene expression analysis, enzyme activity measurements, TAG content determination, and Oil Red O staining.

### Hematological Analysis and TAG Content Determination of Tissues

The concentrations of glucose, TAG, total cholesterol, high-density lipoprotein (HDL) and low-density lipoprotein (LDL) in the serum samples were determined on an automatic biochemical analyzer (Abbott Aeroset Analyzer, Abbott Laboratories, Abbott Park, IL, United States) by the glucose oxidase, glycerol phosphate oxidase-PAP, cholesterol oxidase, PTA-Mg precipitation, and PVS-precipitation methods, respectively, using commercially available colorimetric kits (Biosino Bio-Technology Science Inc., Beijing, China).

Triacylglycerol content in the hepatopancreas and muscle was determined by the glycerol-3-phosphate oxidase p-aminophenol (GPO-PAP) method using a commercial kit (Nanjing Jiancheng Bioengineering Institute, Nanjing, China).

### Enzymatic Activity Assays for Hepatopancreas and Muscle

The enzymatic activities of LPL, FAS, and CPT1 in the hepatopancreas and muscle were detected spectrophotometrically by commercial kits purchased from Shanghai Enzyme-linked Biotechnology Co., Ltd. (Shanghai, China). Hepatopancreas and muscle samples were homogenized in ice-cold PBS (0.01 M, pH = 7.4) and then centrifuged at 5000 × *g* for 5 min at 4°C. The supernatant was collected and immediately used for enzymatic analyses. The optical density (OD) at 450 nm was recorded within 15 min using a microtiter plate reader (NanoQuant 200, Tecan, Austria). The enzyme activities were expressed as units/mg of soluble protein. The amount of enzyme required to convert 1 μmol of substrate per minute at 37°C was defined as a unit of enzyme activity (U). The soluble protein concentration of homogenates was determined based on the method described by Bradford (1976). All analyses were conducted in triplicate.

### Histological Analysis of Hepatopancreas and Muscle

For histological observation of lipid storage in hepatopancreas and muscle, frozen specimens were sectioned (8 μm) by a cryostat microtome (CryoStar NX50, Thermo, United States). Sections were fixed by using cold 10% neutral-buffered formalin solution for 10 min, stained with Oil Red O, and then prepared for light microscopy at 200 × magnification. Five microscopic fields from each sample were randomly selected for calculation of the areas of the lipid droplets, and the results from the 5 individual observations were then combined for the overall results. The area (%) of lipid droplets in Oil Red O–stained sections were based on the following formula: area of lipid droplets/area of randomly selected field from each sample × 100, and this was quantified by Image-Pro Plus 6.0 according to previous reports (Mehlem et al., 2013).

### mRNA Expression Analysis by Quantitative Real-Time PCR

The total RNA isolation and reverse transcription of each sample were conducted based on the methods described by Du et al. (2018). Before RNA extraction, hepatopancreas and muscle tissues were thawed on ice. Total RNA was isolated from these tissues using RNAiso Plus Kits (Takara, Dalian, China) based on the manufacturer’s instructions. The total RNA was dissolved in 50–100 μL RNase-free water, examined on 1% agarose gel, and quantified with a BioTek Synerg^TM^ 2 Multi-detection Microplate Reader (BioTek Instruments, United States). 1 μg of total RNA was used for reverse transcription in a 20-μL reaction volume following the manufacturer’s recommendation for the PrimeScript^TM^ RT reagent Kit (Takara, Dalian, China). The mRNA expression analysis was performed with a quantitative real-time PCR (qPCR) method. All the gene-specific primer pairs (Table 2) were designed using Primer Premier 6.0 software based on the published sequences in the NCBI database and the transcriptome sequences of our lab. The qPCR cycling program consisted of an initial pre-denaturation at 95°C for 30 s and 40 cycles at 95°C for 5 s, 57°C for 10 s, and 72°C for 30 s. Melting curves were monitored systematically, and temperature gradually increased from 55 to 94°C by 0.5°C/s. The amplification efficiency of each target gene was approximately equal (98–102%). All reactions were performed in triplicate and verified to have a single product of correct size by agarose gel electrophoresis. In addition, a preliminary experiment was conducted to check the stability of housekeeping genes [β*-actin*, *18S RNA*, beta-2-microglobulin (*b2m*), glyceraldehyde-3-phosphate dehydrogenase (*gapdh*), hypoxanthine-guanine phosphoribosyltransferase (*hprt*), ribosomal protein L7 (*rpl7*), TATA-box-binding protein (*tbp*), and tubulin alpha chain (*tuba*)] across all samples. The results show that the expression of β*-actin* and *ef1*α were the most stable among samples under the experimental conditions, and they were used as reference genes in this study. The relative expression levels of target genes were calculated using the 2^–ΔΔCt^ method (Livak and Schmittgen, 2001).

**TABLE 2:** Primers and annealing temperatures for quantitative real-time PCR* of grass carp hepatopancreas and muscle.

Gene name	Sequences of primers (5′-3′)	Annealing temperature (°C)	Accession number	Amplification efficiency (%)
**β*-actin***			M25013	
Forward	TATGTTGGTGACGAGGCTCA	56		102
Reverse	GCAGCTCGTTGTAGAAGGTG			
***ef1*α**			GQ266394	
Forward	TGACTGTGCCGTGCTGAT	56		105
Reverse	CGCTGACTTCCTTGGTGATT			
***fasn***			MF631003	
Forward	TGTATGCCACCGCTTATTATTCC	56		104
Reverse	TCCTTTGCCCTGAGTGTTGA			
***srebf1***			KJ162572	
Forward	GTAACAACAGTAGCGTCACCTT	55		100
Reverse	CTTCAGCCAGATGTTCTTCCTC			
***acaca***			HM142590	
Forward	GCAACCACATCTTCCTCAACTT	57		103
Reverse	TCCAGGTAGTAGCCACTCTCA			
***lpl***			FJ716100	
Forward	GCAACCACATCTTCCTCAACTT	56.5		102
Reverse	TCCAGGTAGTAGCCACTCTCA			
***cd36***				
Forward	TTGTGGATGTGGAACCGATTAC	53	KU821103	106
Reverse	CAGGACTGCCGTCTCATTCA			
***pgd***			KP148259	
Forward	ATGAAGGATGTGCTGTGTAT	54		107
Reverse	CGCTGTCTCTGATCTTGG			
***g6pd***			KP148257	
Forward	GAAGGTGGTAGACTCTGAAG	54.5		98
Reverse	CTTGGTGACATCGTGGTAA			
***cpt1a***			KJ816747	
Forward	CAGACACATCGCCGTATT	56		102
Reverse	TTCCACAGCATCCAGAGA			
***lipe***			HQ446238	
Forward	GAGTTCCAATCGCCAGAC	54		101
Reverse	GCCAATGAGTAATCCACAGA			
***pnpla2***				
Forward	CGTTATGTGGATGGTGGAA	55	HQ845211	98
Reverse	TGCCTTGCTCAGTCTGTA			
***ppara***				
Forward	ACAGGCAAGACCAGCACTC	57	FJ595500	102
Reverse	CCACCGAGGCATACTGACA			
***Pparg***				
Forward	CGAGTTCTCCGTCAAGTTCAA	57	EU847421	104
Reverse	CGCAGGTCCGTCATCTTCT			
***fatp1***				
Forward	ACAGCCGATATTACAGGATTGC	55	MK929571	101
Reverse	TCTTCTTGACCACCACAGTTATAC			
***fatp4***				
Forward	CGTCAATCAATCAGCCACTAATAAG	56	MK929565	100
Reverse	CCACTCCATACACCACCACAT			
***fatp6***				
Forward	CTTCAGGACCACAGAGACTTC	55	MK929572	99
Reverse	AGCACAACAGCAGCCATTC			
***acsl1***				
Forward	GTAATGAATATGCTCGTCGGTCTA	54	MK929568	108
Reverse	CCTCTGTGGAGTAATGCTGAAC			
***acsl4***				
Forward	TCTGCTGTCCTGTTGGTCAA	56	MK929567	101
Reverse	AGTCCTCATTATTACGGCTCTCA			
***Vldlr***				
Forward	CAAGTGTCGGAGTGGAGAGT	55	MK929569	105
Reverse	GTCAGGTCACGGCAGATGT			
***lrp1***				
Forward	CACCACACCTCATAACAAGAAC	55	MK929570	101
Reverse	GAGAGCCTTCCAACAATATAGC			
***fabp10***				
*Forward*	AACGGCAACGACTTCATCATC	56	EU220990	102
Reverse	ATCATGGTGGTTCCTCCTATTGT			
***fabp11***				
Forward	GAAGGCTGTAGGTGCTGGTT	54	MK929566	106
Reverse	CTGTCATCTGCTGTTGTCTCATC			
***apob***				
Forward	TCAAGTTGGCAGTTACAGATAGC	55	MK929573	98
Reverse	TAAGGTGGCAGTGGCAGAG			

*fasn, fatty acid synthase; *srebf1*, sterol-regulator element-binding protein; *acaca*, acetyl-CoA carboxylase alpha; *lpl*, lipoprotein lipase; *cd36*: CD36 molecule; *pgd*, phosphogluconate dehydrogenase; *g6pd*, glucose-6-phosphate dehydrogenase; *cpt1a*, carnitine palmitoyltransferase 1A; *lipe*, lipase E, hormone sensitive type; *pnpla2*, patatin-like phospholipase domain-containing protein 2; *ppara*: peroxisome proliferator-activated receptor alpha; *pparg*: peroxisome proliferator-activated receptor gamma; *fatp1*, long-chain fatty acid transport protein 1; *fatp4*, long-chain fatty acid transport protein 4*; fatp6*, long-chain fatty acid transport protein 6; *fabp10*, fatty acid binding protein 10; *fabp11*, fatty acid binding protein 11; *acsl1*, acyl-CoA synthetase long chain family member 1; *acsl4*, acyl-CoA synthetase long chain family member 4; *vldlr*, very low-density lipoprotein receptor; *lrp1*, low-density lipoprotein receptor-related protein; *apob*, apolipoprotein B.*

### Statistical Analysis

Data are presented as mean ± standard error (SE) of means. All data were subjected to one-way ANOVA to test the effect of the four-diet treatment in a fixed effect model. Normality of distribution by the Kolmogorov–Smirnov test and homogeneity of variance by Levene’s test were conducted before fitting the linear model. When the effect of diet treatment was significant, differences between the group means were compared by Duncan’s multiple range test. Statistical analyses were performed using SPSS 19 software (IBM, Armonk, NY, United States). The statistically significant level was set at *P* < 0.05.

## Results

### AELL Effects on Serum Biochemical Indices

The serum TAG content of grass carp decreased gradually as the dietary AELL level increased (*P* < 0.05) (Table 3). The glucose content in serum exhibited no significant differences between the AELL7, AELL14, and AELL21 groups, but the glucose levels in the AELL7 and AELL14 groups were lower than in the control group (*P* < 0.05). The cholesterol content in the AELL14 and AELL21 groups was lower than that in the control and AELL7 groups (*P* < 0.05). The HDL content in the AELL21 group was higher than that in other groups (*P* < 0.05), and the LDL content in the AELL14 and AELL21 groups was lower than that in the control and AELL7 groups (*P* < 0.05).

**TABLE 3:** Effects of dietary alcoholic extract of lotus leaf (AELL) on serum biochemical parameters of juvenile grass carp.

Index	Dietary lotus leaf extract levels^4^								
	AELL0		AELL7		AELL14		AELL21		*P*-values
	Mean	SEM	Mean	SEM	Mean	SEM	Mean	SEM	
TAG^1^ (mmol/L)	2.51^c^	0.08	1.64^b^	0.03	1.49^ab^	0.07	1.36^a^	0.03	0.000
Glucose (mmol/L)	4.43^b^	0.23	3.40^a^	0.29	3.46^a^	0.18	3.81^ab^	0.09	0.031
Cholesterol (mmol/L)	5.52^b^	0.23	5.48^b^	0.09	4.43^a^	0.05	4.69^a^	0.07	0.001
HDL^2^ (mmol/L)	0.84^a^	0.01	0.84^a^	0.02	0.92^a^	0.06	1.07^b^	0.01	0.002
LDL^3^ (mmol/L)	0.99^b^	0.03	1.09^b^	0.03	0.73^a^	0.08	0.82^a^	0.02	0.002

*All data are expressed as means and standard errors (*n* = 9, three replicate tanks, three fish were sampled from each tank). Means with unlike superscript letters were significantly different between the different dietary AELL groups (P < 0.05). ^1^TAG, triacylglycerol. ^2^HDL, high-density lipoprotein. ^3^LDL, low-density lipoprotein. ^4^AELL levels: 0 = 0 AELL supplemented diet, 7 = 0.07% AELL supplemented diet, 14 = 0.14% AELL supplemented diet, 21 = 0.21% AELL supplemented diet.*

### Dietary AELL Effects on Lipid Content and Enzyme activities

**FIGURE 1:**
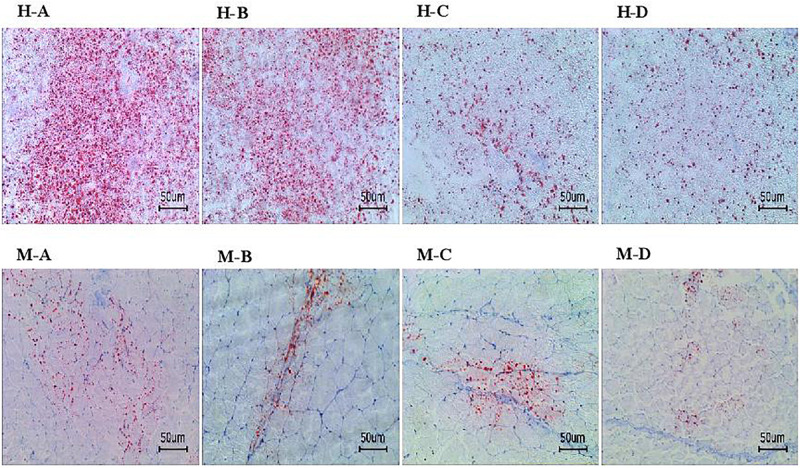
Hepatopancreas and muscle histochemistry (Oil-Red O staining) of grass carp fed with graded levels of dietary alcoholic extract of lotus leaf (AELL). Magnification × 200, the bar means 50 um. H-A, H-B, H-C, H-D: hepatopancreatic histochemistry of group AELLO, AELL7, AELL14, and AELL21, respectively; M-A, M-B, M-C, M-D: muscle histochemistry of group AELLO, AELL7, AELL14, and AELL21, respectively. AELLO = 0 AELL supplemented diet, AELL7 = 0.07% AELL supplemented diet, AELL 14 = 0.14% AELL supplemented diet, AELL21 = 0.21% AELL supplemented diet.

**FIGURE 2:**
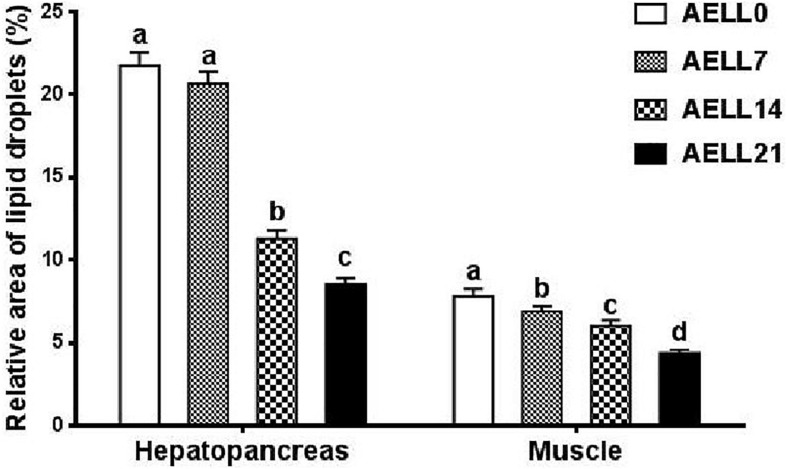
Relative area of lipid droplets in Oil-Red O stained sections of hepatopancreas and muscle of glass carp fed with graded levels of dietary alcoholic extract of lotus leaf (AELL). AELLO = 0 AELL supplemented diet, AELL7 = 0.07% AELL supplemented diet, AELL 14 = 0.14% AELL supplemented diet, AELL21 = 0.21% AELL supplemented diet. Values are expressed as means ± SE (*n* = 9). Different letters over columns indicate significant differences amongst groups (*P* < 0.05, Duncan’s test) after one-way ANOVA with *P* values at 0.000 (hepatopancreas) and 0.000 (muscle), respectively.

**FIGURE 3:**
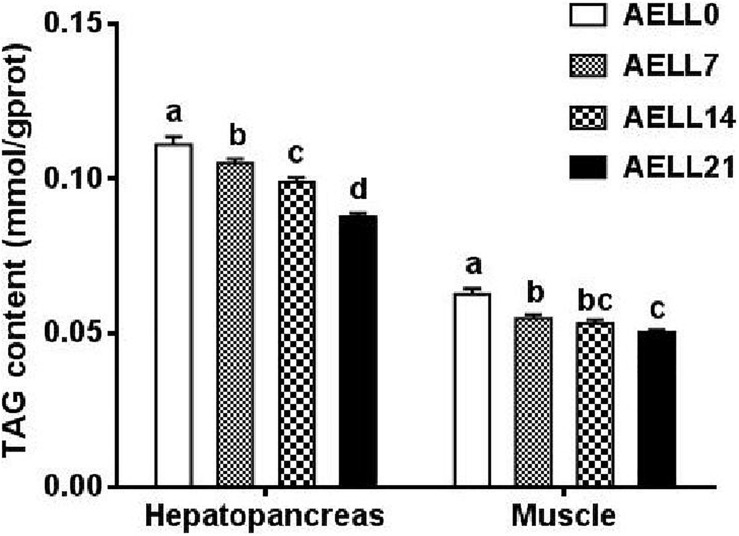
TAG content in hepatopancreas and white muscle of grass carp fed with graded levels of dietary alcoholic extract of lotus leaf (AELL). AELLO = 0 AELL supplemented diet, AELL7 = 0.07% AELL supplemented diet, AELL 14 = 0.14% AELL supplemented diet, AELL21 = 0.21% AELL supplemented diet. Values are expressed as means + SE (*n* = 9, three replicate tanks, three fish were sampled for each tank). Different letters over columns indicate significant differences amongst groups (*P* < 0.05, Duncan’s test) after one-way ANOVA with *P* values at 0.000 (hepatopancreas) and 0.000 (muscle), respectively.

Based on the Oil Red O–stained hepatopancreas and muscle sections, the lipid content in the hepatopancreas and muscle decreased significantly as dietary AELL levels increased (Figures 1, 2). Similarly, the TAG content in the hepatopancreas and muscle decreased significantly as dietary AELL levels increased (Figure 3).

**FIGURE 4:**
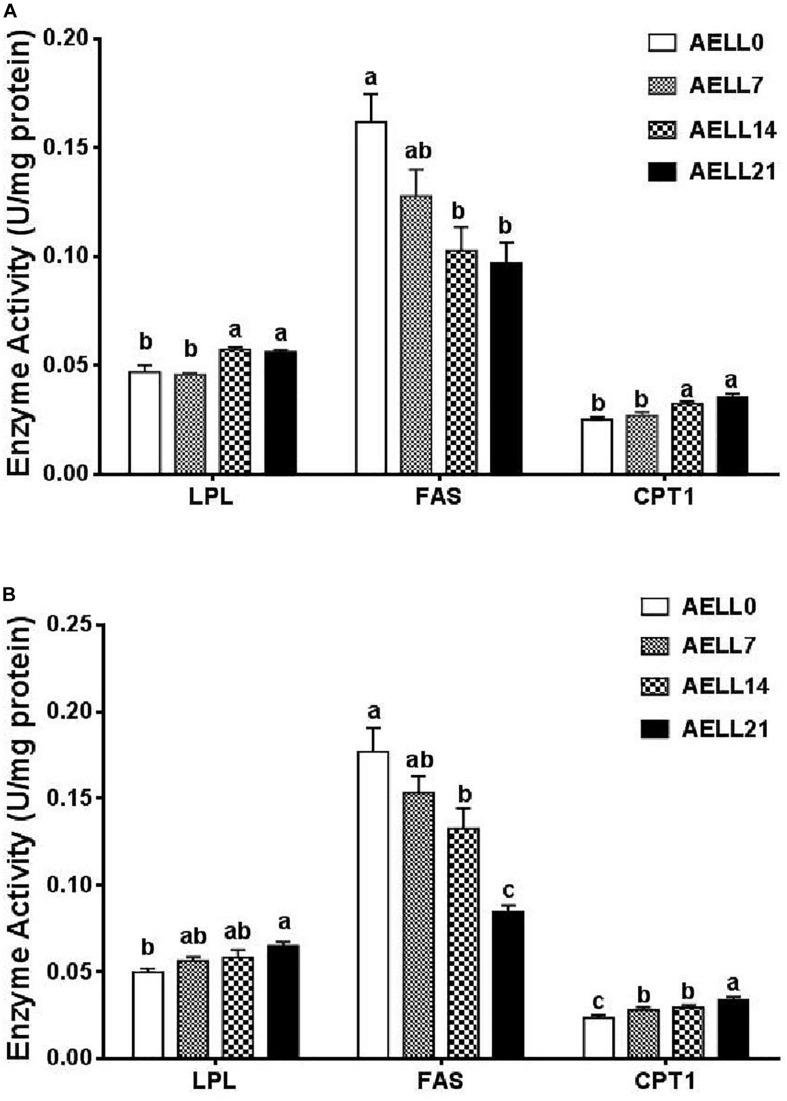
Effect of graded levels of dietary alcoholic extract of lotus leaf (AELL) on enzyme activities involved in lipid metabolism in the hepatopancreas **(A)** and white muscle **(B)** of juvenile glass caip. AELLO = 0 AELL supplemented diet, AELL7 = 0.07% AELL supplemented diet, AELL 14 = 0.14% AELL supplemented diet, AELL21 = 0.21% AELL supplemented diet. Values are expressed as means ± SE (*n* = 9, three replicate tanks, three fish were sampled for each tank). Different letters over columns indicate significant differences among groups (*P* < 0.05) after one-way ANOVA with *P* values at 0.000 (LPL), 0.000 (FAS), and 0.000 (CPT1) in panel **(A)** as well as 0.001 (LPL), 0.000 (FAS), and 0.001 (CPT1) in panel **(B)**, respectively LPL, lipoprotein lipase; FAS, fatty acid synthase; CPT1, carnitine palmitoyltransferase I.

In the hepatopancreas, the LPL and CPT1 enzyme activities tended to increase with dietary AELL levels, and both activities were lower in the AELL0 and AELL7 groups than in the AELL14 and AELL21 groups (*P* < 0.05). Correspondingly, FAS activity decreased as the dietary AELL levels increased, and the AELL0 group had significantly greater FAS activity compared to the AELL14 and AELL21 groups (Figure 4A).

In the muscle, the LPL and CPT1 enzyme activities also increased with the dietary AELL levels (*P* < 0.05). Again, the FAS activity decreased significantly as the dietary AELL levels increased (Figure 4B).

### Dietary AELL Effects on mRNA Expression

**FIGURE 5:**
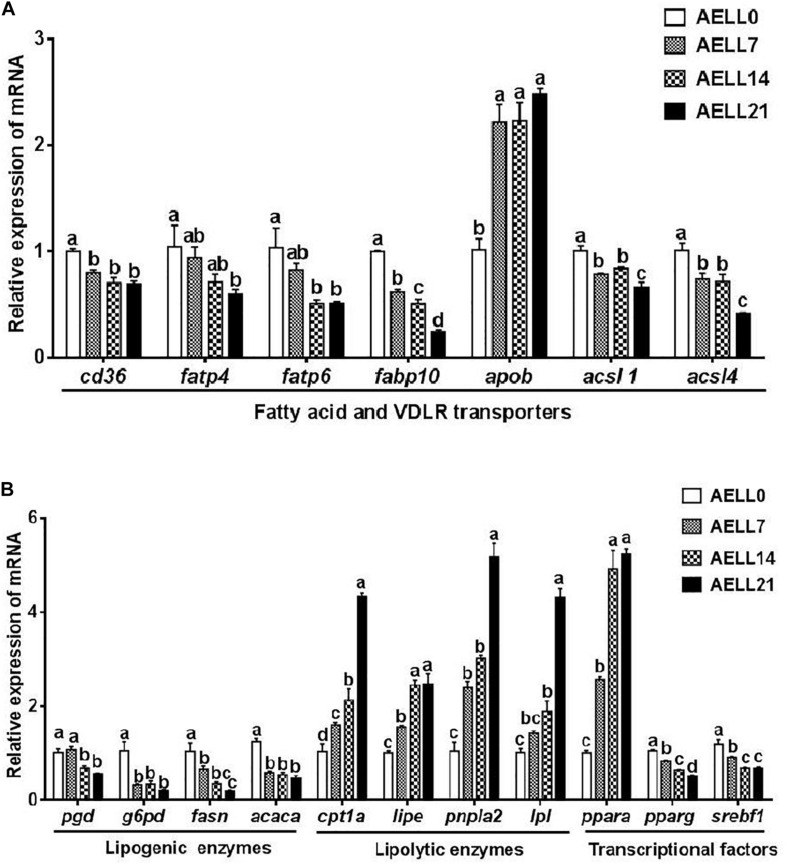
Effect of graded levels of dietary alcoholic extract of lotus leaf (AELL) on the mRNA levels of genes involved in lipid metabolism in the hepatopancreas of juvenile grass carp. AELLO = 0 AELL supplemented diet, AELL7 = 0.07% AELL supplemented diet, AELL 14 = 0.14% AELL supplemented diet, AELL21 = 0.21% AELL supplemented diet. **(A)** Genes involved in fatty acid uptake and transport about lipid metabolism; **(B)** Genes involved in fatty acid synthesis, catabolism and transcription. Values are expressed as means ± SE (*n* = 9, three replicate tanks, three fish were sampled for each tank). Different letters over columns indicate significant differences among groups (*P* < 0.05, Duncan’s test). In panel **(A)**, *P* values for one-way ANOVA are 0.000 (*cd36*), 0.001 (*fatp4*), 0.002 (*fatp6*), 0.015 (*fabplO*), 0.000 (*apob*), 0.001 (*acsll*), and 0.001 (*acsl4*), respectively. In panel **(B)**, *P* values for one-way ANOVA are 0.001 (*pgd*), 0.002 (*g6pd*), 0.002 (*fas*), 0.000 (*acaca*), 0.000 (*cptla*), 0.000 (*lipe*), 0.000 (*pnpla2*), 0.000 (*Ipl*), 0.000 (*ppara*), 0.000 (*pparg*), and 0.001 (*srebfl*), respectively. *cd36*: CD36 molecule; *fap4*, long-chain fatty acid transport protein 1; *fatp6*, long-chain fatty acid transport protein 4; *fabplO*, fatty acid binding protein 11; *apob*, apolipoprotein B; *acsll*, acyl-CoA synthetase long chain family member 1; *acsl4*, acyl-CoA synthetase long chain family member 4; *pgd*, 6-phosphogluconate dehydrogenase; *gpd*, glucose-6-phosphate dehydrogenase,ya.OT, fatty acid synthase; *acaca*, acetyl-CoA carboxylase alpha; *cptla*, camitine palmitoyltransferase 1A; *lipe*, lipase E, hormone sensitive type; *pnplal*, patatin like phospholipase domain-containing protein 2; *Ipl*, lipoprotein lipase, *ppara*: peroxisome proliferator-activated receptor alpha; *pparg*, peroxisome proliferator-activated receptor gamma; *serbfl*, sterol regulator element binding transcription factor 1.

The *cd36* expression in the hepatopancreas was significantly higher in the AELL0 group than in the other groups, but there was no significant difference between the other three groups (Figure 5A). The relative mRNA levels of *fatps* (*fatp4*, *fatp6*) and *fabp10* decreased with increasing AELL concentration. These genes were downregulated significantly in the AELL21 group compared with the control group. However, *apob* expression was lower in the AELL0 control group than in the other groups (*P* < 0.05). Moreover, *acsl1* and *acsl4* gene expression reduced significantly as AELL levels increased, but there was no difference between the AELL7 and AELL14 groups. The gene expression of the lipogenic genes, including *pgd* and *fasn*, decreased progressively (*P* < 0.05) as dietary AELL levels increased (Figure 5B). The transcriptional levels of *g6pd* and *acaca* were also higher in the AELL0 control group than in the other groups (*P* < 0.05), but there were no differences between the other three groups. The expression of all the lipolytic genes (*cpt1a*, *lipe*, *pnpla2*, and *lpl*) increased significantly with the AELL levels. In addition, the *ppara* expression was upregulated significantly by dietary AELL addition, but there was no difference between the AELL14 and AELL21 groups. In contrast, the expression of the transcription factors *pparg* and *srebf1* decreased gradually as the AELL levels increased (*P* < 0.05) although that *srebf1* gene expression was not different between the AELL14 and AELL21 groups.

**FIGURE 6:**
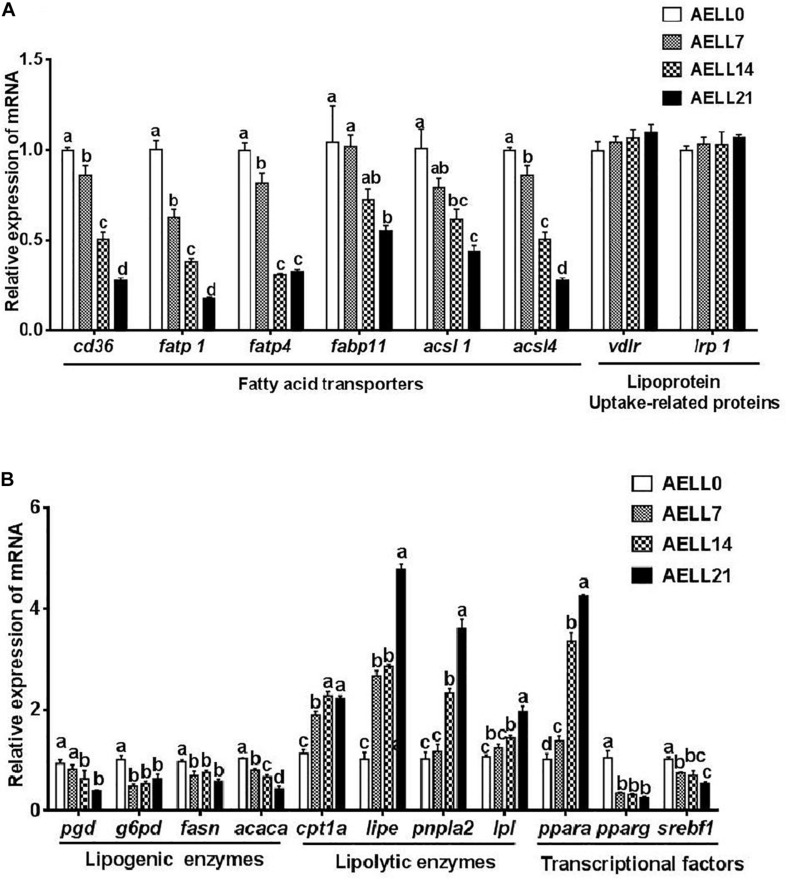
Effect of graded levels of dietary lotus leaf extract (AELL) on the mRNA levels of genes involved in lipid metabolism in the muscle of juvenile grass carp. AELLO = 0 AELL supplemented diet, AELL7 = 0.07% AELL supplemented diet, AELL 14 = 0.14% AELL supplemented diet, AELL21 = 0.21% AELL supplemented diet. **(A)** Genes involved in fatty acid uptake about lipid metabolism; **(B)** Genes involved in fatty acid synthesis, catabolism and transcription. Values are expressed as means ± SE (*n* = 9, three replicate tanks, three fish were sampled for each tank). Different letters over columns indicate significant differences among groups (*P* < 0.05, Duncan’s test). In panel **(A)**, *P* values for one-way ANOVA are 0.000 (*cd36*), 0.000 (*fatpl*), 0.007 (*fatp4*), 0.037 (*fabpll*), 0.002 (*acsll*), 0.000 (*acsl4*), 0.500 (*vldlr*), and 0.730 (*Irpl*), respectively. In panel **(B)**, *P* values for one-way ANOVA are 0.002 (*pgd*), 0.002 (*g6pd*), 0.008 (*fasti*), 0.000 (*acaca*), 0.000 (*cptla*), 0.000 (*lipe*), 0.000 (*pnplal*), 0.000 (*Ipl*), 0.000 (*ppara*), 0.000 (*pparg*), and 0.002 (*srebfl*), respectively. *cd36*: CD36 molecule; *fatpl*, long-chain fatty acid transport protein 1; *fatp4*, long-chain fatty acid transport protein 4; *fabpll*, fatty acid binding protein 11; *acsll*, acyl-CoA synthetase long chain family member 1; *acsl4*, acyl-CoA synthetase long chain family member 4; *vldlr*, very low density lipoprotein receptor; *Irpl*, low-density lipoprotein receptor-related protein; *pgd*, phosphogluconate dehydrogenase; *g6pd*, glucose-6-phosphate dehydrogenase; *fasn*, fatty acid synthase; *acaca*, acetyl-CoA carboxylase alpha; *cptla*, camitine palmitoyltransferase 1A; *lipe*, lipase E, hormone sensitive type; *pnpla2*, patatin like phospholipase domain-containing protein 2; *Ipl*, lipoprotein lipase; *ppara*, peroxisome proliferator-activated receptor alpha; *pparg*, peroxisome proliferator-activated receptor gamma; *serbfl*, sterol regulator element binding transcription factor 1.

The muscle mRNA levels of fatty acid transporters (*cd36*, *fatp1*, *fatp4*, and *fabp11*) and *acsls* (*acsl1* and *acsl4*) decreased progressively as the dietary AELL levels increased (*P* < 0.05) (Figure 6A). However, *vldlr* and *lrp1* expression showed no significant difference among the four groups (*P* > 0.05). The expression of the lipogenic genes, including *pgd* and *acaca*, progressively decreased as the AELL levels increased (*P* < 0.05) (Figure 6B). The transcriptional levels of *g6pd* and *fasn* were significantly higher in the AELL0 group than other groups, but there were no differences between the other three groups. However, mRNA levels of all the lipolytic genes (*cpt1a*, *lipe*, *pnpla2*, and *lpl*) increased with higher AELL levels. In addition, *ppara* expression increased progressively with dietary AELL levels (*P* < 0.05), and the relative mRNA level of *srebf1* decreased. The transcriptional level of *pparg* was significantly higher in the control group than the other groups, but there were no differences between the other three groups.

## Discussion

To our knowledge, this is the first investigation on the effect of dietary AELL on the lipid content of serum, muscle, and hepatopancreas in fish and the related molecular effects. The results show that dietary AELL can decrease serum TAG, cholesterol, and LDL levels and increase the serum HDL level of grass carp. Similar hypolipidemic effects of dietary lotus leaf extract addition are reported in hamsters and rats (Du et al., 2010; Lee and Lee, 2011; Guo et al., 2013; Lee et al., 2015). Thus, dietary lotus leaf extract can improve the blood lipid profiles of animals for better health.

Apart from the hypolipidemic effect, dietary supplementation of lotus leaf extract is reported to decrease hepatic TAG in rats and hamsters (Du et al., 2010; Lee and Lee, 2011; Guo et al., 2013). Our study is in agreement with these results. Herein, the TAG content and lipid droplets in the carp hepatopancreas and muscle declined as dietary AELL increased. Given the decrease in lipid within the serum, muscle, and hepatopancreas of the grass carp in this study, the dietary AELL appears to be preventing lipid deposition in the hepatopancreas and muscle of grass carp by modulating the serum lipid and lipoprotein concentrations. Furthermore, such an effect of the lotus leaf extract may suppress adipose tissue differentiation and reduce triacylglyceride accumulation (Siegner et al., 2010).

The mechanism by which dietary AELL inclusion reduces lipid deposition was explored in gene expression experiments in the present study. In the circulatory system, TAG, cholesterol, free fatty acids, and lipoproteins are transported to different tissues for metabolism. When fatty acids are absorbed into tissues, fatty acid transporters, such as fatty acid translocase/CD36, FABPs, and FATPs, are key contributors to the transmembrane process (Sclafani et al., 2007). Overexpression of FATPs and long-chain acyl-CoA synthetase (ACSL) lead to a significant increase in acyl-CoA synthetase activity as well as the uptake of fatty acids (Krammer et al., 2011). In this study, the mRNA levels of fatty acid transporters, such as *cd36*, *fabps*, *fatps*, and *acsls*, decreased significantly as AELL levels increased, which indicates that AELL might result in the suppression of fatty acid transportation and absorption into hepatopancreas and muscle. Similar results regarding lotus leaf extract regulating the gene expression of fatty acid transporters are reported in hamsters (Guo et al., 2013). Interestingly, in the present study, *fatp6* and *fabp10* gene expression were high in the hepatopancreas but very low in muscle. In contrast, *fatp1* and *fabp11* gene expression were high in muscle but low in the hepatopancreas. The differential expression of these genes in the hepatopancreas and muscle indicate potential tissue-specific absorption of fatty acids by different tissues (Saxena et al., 1992). Similar differential gene expression patterns of these fatty acid transporters are reported in Atlantic salmon (*Salmo salar L.*) (Torstensen et al., 2009) and hybrid grouper (*Epinephelus lanceolatus*♂× *Epinephelus fuscoguttatus*♀) (Tan et al., 2019). In mammals, *fatp1* and *fatp4* are expressed highly in adipose tissue, brain, liver, skin, and heart (Doege and Stahl, 2006). Moreover, APOB is a key regulator of newly synthesized TAG transportation from the liver to the peripheral tissues via VLDL (Kawano and Cohen, 2013). The increase in *apob* expression might suggest that AELL addition stimulates hepatic lipid export and reduces lipid accumulation in the hepatopancreas. In summary, AELL supplementation resulted in the inhibition of TAG accumulation in the muscle and liver by limiting the lipid absorption into tissues and promoting the export of newly synthesized TAG from the liver.

It is demonstrated that *pgd*, *g6pd*, *fasn*, and *acaca* are regulatory genes in *de novo* lipogenesis, and FAS is a key enzyme in this process (Kawano and Cohen, 2013). This study reveals that dietary AELL supplementation decreased FAS activity and downregulated the gene expression of *pgd*, *g6pd*, *fasn*, and *acaca* in both the hepatopancreas and muscle, which indicates that dietary supplementation of AELL reduces lipid deposition in the hepatopancreas and muscle of juvenile grass carp via the reduction of fatty acid synthesis. Similarly, in hamsters, the addition of lotus leaf extract to the diet decreased the mRNA levels of lipogenesis-related genes and significantly decreased lipid content in the liver and plasma (Guo et al., 2013). Other flavonoid-rich plant extracts have shown similar regulation of lipid metabolism (Madushani Herath et al., 2018; Veeramani et al., 2018). Moreover, LPL is the principal enzyme to hydrolyze lipoproteins within the cells and to supply free fatty acids for further lipid metabolism (Nilsson-Ehle et al., 1980). The supplementation of AELL in this study stimulated LPL activity and upregulated *lpl* gene expression in the hepatopancreas and muscle, resulting in the enhancement of lipoprotein hydrolysis. Furthermore, *pnpla2*, *cpt1a*, and *lipe* are the main genes involved in fatty acid β-oxidation (Kawano and Cohen, 2013). ATGL (encoded by *pnpla2*) plays an important role in the initial step of TAG hydrolysis, and CPT1 catalyzes the rate-limiting step in lipid catabolism (Kawano and Cohen, 2013). The present study indicates that dietary AELL addition stimulates CPT1 activity and upregulates the mRNA levels of *pnpla2*, *cpt1a*, and *lipe* in both the hepatopancreas and muscle. These results might suggest that dietary AELL addition exhibits a strong beta oxidation stimulating effect. Guo et al. (2013) also report that the addition of lotus leaf extract to the diet increased the mRNA levels of genes involved in fatty acid β-oxidation in the liver of hamsters as observed in the present study. Other flavonoid-rich plant extracts also show similar regulation of lipid catabolism (Liu et al., 2017; Dong et al., 2018). These results indicate that AELL, like other flavonoid-rich plant extracts, have positive effects on lipid catabolism through enhancement of fatty acid β-oxidation.

Previous studies indicate that genes in lipid metabolic pathways are regulated by *srebf1*, *ppara*, and *pparg* at the transcription level (Kawano and Cohen, 2013). During lipid metabolism, PPARA works as a ligand-activated transcription factor to activate lipolytic genes by binding to corresponding response elements (Minghetti et al., 2011). In the present study, the upregulation of *ppara* expression in the hepatopancreas and muscle, together with the increased expression of lipolytic genes (*pnpla2*, *cpt1a*, and *lipe*), suggest that dietary AELL might suppress lipid accumulation by enhancing lipolysis with the participation of *ppara*. In addition, the upregulation of *ppara* expression corresponding to the increased *cpt1a* expression in the liver was also observed in hamsters fed a high dosage of nuciferine in their diet (Guo et al., 2013). Lipogenesis is controlled primarily at the transcriptional level, and SREBF1 is the transcription factor that promotes the expression of many lipogenic genes, including *fasn* and *acaca* (Csaki and Reue, 2010). It is demonstrated that *srebf1* and *pparg* regulate lipogenic gene expression so as to mediate TAG synthesis and lipid accumulation (Amemiya-Kudo et al., 2002; Barish, 2006). Hence, the reduction in lipid deposition of both hepatopancreas and muscle by dietary AELL supplementation in this study may be partly due to the inhibition of lipogenesis through the downregulation of the expression of both *srebf1* and *pparg*, followed by the decrease in FAS activity and mRNA levels of *pgd*, *g6pd*, and *fasn*. Similar pathways of lipid metabolism and their regulatory mechanisms have been elucidated in hamsters (Guo et al., 2013), hybrid grouper (Tan et al., 2019), and yellow catfish (*Pelteobagrus fulvidraco*) (Zheng et al., 2015). In this regard, the present study indicates that dietary AELL may regulate lipid metabolism and deposition via transcription factors (*srebf1*, *ppara*, and *pparg*) in the hepatopancreas and muscle of grass carp.

To conclude, the dietary supplementation of AELL shows a positive effect on serum lipid content, suppressed fatty acid lipogenesis, and enhanced lipid catabolism and exportation. Thus, the TAG accumulation is inhibited in the hepatopancreas and muscle of grass carp in a dose-dependent manner. These results reveal similar regulatory mechanisms of dietary AELL on the lipid metabolism in both the hepatopancreas and muscle of juvenile grass carp. The results of this study provide new insights into the application of AELL as a feed additive in aquaculture for the control of fish health and meat quality.

## Data Availability Statement

The datasets presented in this study can be found in online repositories. The names of the repository/repositories and accession number(s) can be found in the article/supplementary material.

## Ethics Statement

The animal study was reviewed and approved by the Institutional Animal Care and Use Committee (IACUC) of Huazhong Agricultural University (Wuhan, China).

## Author Contributions

QT and JY designed the experiment and analyzed the data. JY conducted the sample analysis and drafted the manuscript. PH and YZ conducted the feeding trial. YX helped with the analysis of gene expression data. QT and XL revised the manuscript. All authors approved the final manuscript.

## Conflict of Interest

The authors declare that the research was conducted in the absence of any commercial or financial relationships that could be construed as a potential conflict of interest.

## References

[B1] Agnihotri V. K. ElSohly H. N. Khan S. I. Jacob M. R. Joshi V. C. Smillie T. (2008). Constituents of Nelumbo nucifera leaves and their antimalarial and antifungal activity. *Phytochem. Lett.* 1 89–93. 10.1016/j.phytol.2008.03.003 29152009 PMC5690537

[B2] Agrawal A. (2011). Pharmacological activities of flavonoids: a review. *Int. J. Pharm. Sci. Nanotechnol.* 4 1394–1398. 10.18468/estcien.2017v7n3.p29-35

[B3] Ahn J. H. Kim E. S. Lee C. Kim S. Cho S. H. wang B. Y. (2013). Chemical constituents from Nelumbo nucifera leaves and their anti-obesity effects. *Bioorg. Med. Chem. Lett.* 23 3604–3608. 10.1016/j.bmcl.2013.04.013 23642481

[B4] Amemiya-Kudo M. Shimano H. Hasty A. H. Yahagi N. Yoshikawa T. Matsuzaka T. (2002). Transcriptional activities of nuclear SREBP-1a,-1c, and-2 to different target promoters of lipogenic and cholesterogenic genes. *J. Lipid Res.* 43 1220–1235. 10.1194/jlr.M100417-JLR200 12177166

[B5] Barish G. D. (2006). Peroxisome proliferator-activated receptors and liver X receptors in atherosclerosis and immunity. *J. Nutr.* 136 690–694. 10.1093/jn/136.3.690 16484544

[B6] Bradford M. M. (1976). A rapid and sensitive method for the quantitation of microgram quantities of protein utilizing the principle of protein-dye binding. *Anal. Biochem.* 72 248–254. 10.1016/0003-2697(76)90527-3 942051

[B7] Chen Y. Li Q. Kuang Z. P. Zhao X. Yi R. K. He X. W. (2020). Inhibitory effect of flavonoid extract of lotus leaf on alcohol-induced gastric injury by antioxidant capacity in mice. *J. Food Qual.* 2020:11 10.1155/2020/1206247

[B8] Chen Y. Varghese Z. Ruan X. Z. (2014). The molecular pathogenic role of inflammatory stress in dysregulation of lipid homeostasis and hepatic steatosis. *Genes Dis.* 1 106–112. 10.1016/j.gendis.2014.07.007 30258859 PMC6150078

[B9] Csaki L. S. Reue K. (2010). Lipins: multifunctional lipid metabolism proteins. *Annu. Rev. Nutr.* 30 257–272. 10.1146/annurev.nutr.012809.104729 20645851 PMC3738581

[B10] Ding Y. Pu L. Kan J. (2017). Hypolipidemic effects of lipid-lowering granulated tea preparation from *Monascus*-fermented grains (adlay and barley bran) mixed with lotus leaves on Sprague-Dawley rats fed a high-fat diet. *J. Funct. Foods* 32 80–89. 10.1016/j.jff.2017.02.025

[B11] Doege H. Stahl A. (2006). Protein-mediated fatty acid uptake: novel insights from in vivo models. *Physiology* 21 259–268. 10.1152/physiol.00014.2006 16868315

[B12] Dong L. Han X. Tao X. Xu L. Xu Y. Fang L. (2018). Protection by the total flavonoids from *Rosa laevigata* Michx fruit against lipopolysaccharide-induced liver injury in mice via modulation of FXR signaling. *Foods* 7:88. 10.3390/foods7060088 29890650 PMC6025249

[B13] Du H. Yao J. Zhou H. Leng X. Wu J. He S. (2018). Optimal dietary lipid level promoted ovary development of Chinese sturgeon (*Acipenser sinensis*) broodstocks. *Aquaculture* 495 288–294. 10.1016/j.aquaculture.2018.05.046

[B14] Du H. You J. S. Zhao X. Park J. Y. Kim S. H. Chang K. J. (2010). Antiobesity and hypolipidemic effects of lotus leaf hot water extract with taurine supplementation in rats fed a high fat diet. *Biomed. Sci.* 17(Supp. 1):S42. 10.1186/1423-0127-17-S1-S42 PMC2994410 20804619

[B15] Duque-Guimarães D. E. de Castro J. Martinez-Botas J. Sardinha F. L. Ramos M. P. Herrera E. (2009). Early and prolonged intake of partially hydrogenated fat alters the expression of genes in rat adipose tissue. *Nutrition* 25 782–789. 10.1016/j.nut.2008.12.004 19251397

[B16] FAO (2018). *FAO yearbook: Fishery and Aquaculture Statistics 2016.* Rome: FAO Fisheries and Aquaculture Department.

[B17] Gui J. Zhu Z. (2012). Molecular basis and genetic improvement of economically important traits in aquaculture animals. *Chin. Sci. Bull.* 57 1751–1760. 10.1007/s11434-012-5213-0

[B18] Guo F. Yang X. Li X. Feng R. Guan C. Wang Y. (2013). Nuciferine prevents hepatic steatosis and injury induced by a high-fat diet in hamsters. *PLoS One* 8:e63770. 10.1371/journal.pone.0063770 23691094 PMC3655021

[B19] Hoseinifar S. H. Sun Y. Z. Zhou Z. Z. Doan H. V. Davies S. J. Harikrishnan R. (2020). Boosting Immune function and disease bio-control through environment-friendly and sustainable approaches in finfish aquaculture: herbal therapy scenarios. *Rev. Fish. Sci. Aquac.* 28 303–321. 10.1080/23308249.2020.1731420

[B20] Kawano Y. Cohen D. E. (2013). Mechanisms of hepatic triglyceride accumulation in non-alcoholic fatty liver disease. *J. Gastroenterol.* 48 434–441. 10.1007/s00535-013-0758-5 23397118 PMC3633701

[B21] Kerner J. Hoppel C. (2000). Fatty acid import into mitochondria. *Biochim. Biophys. Acta. Mol. Cell. Biol. Lipids* 1486 1–17. 10.1016/S1388-1981(00)00044-5 10856709

[B22] Kim M. Pichiah P. B. T. Kim D. K. Cha Y. S. (2017). Black adzuki bean (*Vigna angularis*) extract exerts phenotypic effects on white adipose tissue and reverses liver steatosis in diet-induced obese mice. *J. Food Biochem.* 41:e12333 10.1111/jfbc.12333

[B23] Kim S. M. Yun H. J. Yi H. S. Won C. W. Kim J. E. Park S. D. (2009). Nelumbo nucifera leaves inhibit HASMC proliferation and migration activated by TNF-alpha. *Korea J. Herbol.* 24 77–86.

[B24] Krammer J. Digel M. Ehehalt F. Stremmel W. Füllekrug J. Ehehalt R. (2011). Overexpression of CD36 and acyl-CoA synthetases FATP2, FATP4 and ACSL1 increases fatty acid uptake in human hepatoma cells. *Int. J. Med. Sci.* 8 599–614. 10.7150/ijms.8.599 22022213 PMC3198256

[B25] Lee K. Kim J. Lee N. Park S. Cho H. Chun Y. (2015). Effects of potato and lotus leaf extract intake on body composition and blood lipid concentration. *J. Exerc. Nutr. Biochem.* 19 25–30. 10.5717/jenb.2015.19.1.25 25960952 PMC4424443

[B26] Lee K. S. Lee K. Y. (2011). Effect of lotus (*Nelumbo nucifera*) leaf extract on serum and liver lipid levels of rats fed a high fat diet. *J. Korean Soc. Food Sci. Nutr.* 40 1544–1547. 10.3746/jkfn.2011.40.11.1544

[B27] Liu C. Ma J. Sun J. Cheng C. Feng Z. Jiang H. (2017). Flavonoid-rich extract of *Paulownia fortunei* flowers attenuates diet-induced hyperlipidemia, hepatic steatosis and insulin resistance in obesity mice by AMPK pathway. *Nutrients* 9:959. 10.3390/nu9090959 28867797 PMC5622719

[B28] Livak K. J. Schmittgen T. D. (2001). Analysis of relative gene expression data using real-time quantitative PCR and the 2^–ΔΔCT^ method. *Methods* 25 402–408. 10.1006/meth.2001.1262 11846609

[B29] Ma W. Lu Y. Hu R. Chen J. Zhang Z. Pan Y. (2010). Application of ionic liquids based microwave-assisted extraction of three alkaloids N-nornuciferine. O-nornuciferine, and nuciferine from lotus leaf. *Talanta* 80 1292–1297. 10.1016/j.talanta.2009.09.027 20006090

[B30] Madushani Herath K. H. I. N. Cho J. Kim A. Eom T. K. Kim J. S. Kim J. B. (2018). Phenolic acid and flavonoid-rich fraction of *Sasa quelpaertensis* Nakai leaves prevent alcohol induced fatty liver through AMPK activation. *J. Ethnopharmacol.* 224 335–348. 10.1016/j.jep.2018.06.008 29906537

[B31] Mehlem A. Hagberg C. E. Muhl L. Eriksson U. Falkevall A. (2013). Imaging of neutral lipids by oil red O for analyzing the metabolic status in health and disease. *Nat. Protoc.* 8 1149–1154. 10.1038/nprot.2013.055 23702831

[B32] Minghetti M. Leaver M. J. Tocher D. R. (2011). Transcriptional control mechanisms of genes of lipid and fatty acid metabolism in the Atlantic salmon (*Salmo salar L.*) established cell line. SHK-1. *Biochim. Biophys. Acta. Mol. Cell Biol. Lipids* 1811 194–202. 10.1016/j.bbalip.2010.12.008 21193059

[B33] Mukherjee P. K. Mukherjee D. Maji A. K. Rai S. Heinrich M. (2009). The sacred lotus (*Nelumbo nucifera*)-phytochemical and therapeutic profile. *J. Pharm. Pharmacol.* 61 407–422. 10.1211/jpp/61.04.0001 19298686

[B34] Munglue P. (2015). Effect of *Nelumbo nucifera* Gaertn. leaf extract on growth performance of Nile tilapia (*Oreochromis niloticus*). *J. Food Health Bioenviron. Sci.* 8 45–56.

[B35] Nilsson-Ehle P. Garfinkel A. S. Schotz M. C. (1980). Lipolytic enzymes and plasma lipoprotein metabolism. *Annu. Rev. Biochem.* 49 667–693. 10.1146/annurev.bi.49.070180.003315 6996570

[B36] Perman J. C. Boström P. Lindbom M. Lidberg U. StÅhlman M. Hägg D. (2011). The VLDL receptor promotes lipotoxicity and increases mortality in mice following an acute myocardial infarction. *J. Clin. Invest.* 121 2625–2640. 10.1172/JCI43068 21670500 PMC3223818

[B37] Saxena U. Klein M. G. Vanni T. M. Goldberg I. J. (1992). Lipoprotein lipase increases low density lipoprotein retention by subendothelial cell matrix. *J. Clin. Invest.* 89 373–380. 10.1172/JCI115595 1737833 PMC442862

[B38] Sclafani A. Ackroff K. Abumrad N. A. (2007). CD36 gene deletion reduces fat preference and intake but not post-oral fat conditioning in mice. *Am. J. Physiol. Regul. Integr. Comp. Physiol.* 293 R1823–R1832. 10.1152/ajpregu.00211.2007 17804586

[B39] Siegner R. Heuser S. Holtzmann U. Söhle J. Schepky A. Raschke T. (2010). Lotus leaf extract and L-carnitine influence different processes during the adipocyte life cycle. *Nutr. Metab.* 7:66. 10.1186/1743-7075-7-66 20687953 PMC2922297

[B40] Song H. Han W. Yan F. Xu D. Chu Q. Zheng X. (2016). Dietary Phaseolus vulgaris extract alleviated diet-induced obesity, insulin resistance and hepatic steatosis and alters gut microbiota composition in mice. *J. Funct. Foods* 20 236–244. 10.1016/j.jff.2015.10.022

[B41] Su Q. Lu Z. Deng Q. Wei B. (2015). Alcoholic extract of lotus leaves improves lipid profile in rats with HIV protease inhibitor-induced dyslipidaemia. *West Indian. Med. J*. 64 195–200. 10.7727/wimj.2014.373 26426169 PMC4763890

[B42] Tan X. Sun Z. Ye C. (2019). Dietary Lycium barbarum extract administration improved growth, meat quality and lipid metabolism in hybrid grouper (*Epinephelus lanceolatus* ♂×*E. fuscoguttatus* ♀) fed high lipid diets. *Aquaculture* 504 190–198. 10.1016/j.aquaculture.2019.01.044

[B43] Tanoli T. Yue P. Yablonskiy D. Schonfeld G. (2004). Fatty liver in familial hypobetalipoproteinemia. *J. Lipid Res.* 45 941–947. 10.1194/jlr.M300508-JLR200 14967820

[B44] Torstensen B. E. Nanton D. A. Olsvik P. A. Sundvold H. Stubhaug I. (2009). Gene expression of fatty acid-binding proteins, fatty acid transport proteins (CD36 and FATP) and β-oxidation-related genes in Atlantic salmon (*Salmo salar L.*) fed fish oil or vegetable oil. *Aquacult. Nutr.* 15 440–451. 10.1111/j.1365-2095.2008.00609.x

[B45] Veeramani C. Alsaif M. A. Al-Numair K. S. (2018). Herbacetin, a flaxseed flavonoid, ameliorates high percent dietary fat induced insulin resistance and lipid accumulation through the regulation of hepatic lipid metabolizing and lipid-regulating enzymes. *Chem. Biol. Interact.* 288 49–56. 10.1016/j.cbi.2018.04.009 29653099

[B46] Wang J. Liang X. F. He S. Li J. Huang K. Zhang Y. P. (2018). Lipid deposition pattern and adaptive strategy in response to dietary fat in Chinese perch (*Siniperca chuatsi*). *Nutr. Metab.* 15:77. 10.1186/s12986-018-0315-6 30410565 PMC6211486

[B47] Zhang D. D. Zhang J. G. Wu X. Liu Y. Gu S. Y. Zhu G. H. (2015). Nuciferine downregulates Per-Arnt-Sim kinase expression during its alleviation of lipogenesis and inflammation on oleic acid-induced hepatic steatosis in HepG2 cells. *Front. Pharmacol*. 6:238. 10.3389/fphar.2015.00238 26539118 PMC4612658

[B48] Zhao H. Chong J. Tang R. Li L. Xia J. Li D. (2018). Metabolomics investigation of dietary effects on flesh quality in grass carp (*Ctenopharyngodon idellus*). *GigaScience* 7 1–18. 10.1093/gigascience/giy111 30192945 PMC6176498

[B49] Zhao X. L. Deng F. M. (2013). Study on variation of flavonoids and nuciferine content of lotus leaves. *Food Machinery* 9 37–40.

[B50] Zheng J. L. Luo Z. Hu W. Liu C. X. Chen Q. L. Zhu Q. L. (2015). Different effects of dietary Zn deficiency and excess on lipid metabolism in yellow catfish *Pelteobagrus fulvidraco*. *Aquaculture* 435 10–17. 10.1016/j.aquaculture.2014.09.011 25722194

[B51] Zhu Y. Hu P. Yao J. Xu D. Xu Y. Tan Q. (2019). Optimal dietary alcoholic extract of lotus leaf improved growth performance and health status of grass carp (*Ctenopharyngodon idellus*). *Fish Shellfish Immunol.* 93 1–7. 10.1016/j.fsi.2019.07.039 31315061

